# Coping with Cognitive Impairment in People with Parkinson's Disease and Their Carers: A Qualitative Study

**DOI:** 10.1155/2018/1362053

**Published:** 2018-04-08

**Authors:** Rachael A. Lawson, Daniel Collerton, John-Paul Taylor, David J. Burn, Katie R. Brittain

**Affiliations:** ^1^Institute of Neuroscience, Newcastle University, Newcastle upon Tyne, UK; ^2^Department of Nursing, Midwifery and Health, Northumbria University, Newcastle upon Tyne, UK

## Abstract

Cognitive impairment is common in Parkinson's disease (PD). However, the psychosocial impact of living and coping with PD and cognitive impairment in people with PD and their carers have not been explored. This paper draws on a qualitative study that explores the subjective impact of cognitive impairment on people with PD and their carers. Thirty-six one-to-one interviews were completed; people with PD were from three groups: normal cognition, mild cognitive impairment, and dementia. Data collection and analysis were iterative, and verbatim transcripts were analysed using thematic analysis. Themes were interpreted in consultation with coping and adaptation theory. The analysis revealed four main themes: threats to identity and role, predeath grief and feelings of loss in carers, success and challenges to coping in people with PD, and problem-focused coping and finding meaning in caring. Our data highlight how cognitive impairment can threaten an individual's self-perception; the ostensible effects of cognitive impairment depended on the impact individual's perceived cognitive impairment had on their daily lives. For carers, cognitive impairment had a greater emotional impact than the physical symptoms of PD. The discussion that developed around protective factors provides possible opportunities for future interventions, such as psychological therapies to improve successful adjustment.

## 1. Introduction

Cognitive impairment in Parkinson's disease (PD) is a common nonmotor symptom, with up to 80% of people with PD developing dementia (PDD) [1]. Previous quantitative studies have shown that cognitive impairment can be detrimental to the quality of life of people with PD and their informal caregivers [2, 3]. However, these studies could not explain the mechanism or what aspects of cognitive impairment affected people with PD and their carers. In-depth qualitative studies may be more suitable to address these questions.

Qualitative aspects of the carer experience associated with dementia have been thoroughly explored [4–10]. Spousal carers of people with dementia or cognitive impairment experience high levels of burden; they have related feeling stressed, anxious and depressed, and neglected self-care [11]. However, few qualitative studies explore the effects of cognitive impairment on people with PD and/or their carers. Birgersson and Edberg [12] more broadly investigated the experience of support in people with PD and their carers. People with PD may have high perceived support and experience a sense of solidarity and community but may also feel humiliated, excluded, or misunderstood. For carers, they may be recognised for the support they give but may themselves feel neglected, uncertain about the future, and isolated [12]. The perception that PD was taking over the person was a theme in a study by Williamson et al. [13], which was similar to findings in other studies [12, 14, 15]. Conversely, Chiong-Rivero et al. [16] found that both carers and people with PD found a new appreciation for life and that meaningful relationships with loved ones were strengthened.

Research into coping and adjustment in the context of chronic illness is heterogeneous; there are multiple complex conceptualisations, theoretical frameworks, contributing factors, and measures of adjustment across chronic illnesses [17–20]. Theories of coping and adjustment postulate that maintaining good quality of life requires successful coping and adjustment skills in response to stressors from a chronic illness. However, not all people with chronic illness successfully adjust; those who do not can experience psychological disturbances such as fear, anger, distress, and depression [17, 18]. Adjustment and coping heavily rely on individuals having the cognitive reserve necessary to implement successful strategies outlined in the models [21]. Hurt et al. [22] found that even mild to moderate cognitive impairment in subjects with PD can contribute to reduced task-oriented coping strategies. Similarly, Kudlicka et al. [23] suggested that executive dysfunction could contribute to ineffective strategies to overcome limitations of PD or ineffective coping strategies relating to psychological distress.

Therefore, PD may affect emotional well being, independence, and sense of self in both people with PD and their carer. Previous research has shown that there are numerous ways to cope with illness-related stressors, but that cognition plays a role in adjustment and cognitive impairment may make successful adjustment more difficult. What is not understood is the role that cognitive impairment associated with PD has, how it is experienced, and how it is managed from both the perspectives of the person with PD and their carer. We sought to understand whether cognitive impairment affected coping with PD and caring for someone with PD, and if so what the impact of cognitive impairment was. We also sought to understand how individuals interpreted cognitive impairment and associated symptoms, such as hallucinations. Finally, we wanted to deepen the understanding of the relationship between carers and people with PD, in terms of their needs and priorities that may be unrecognised.

## 2. Method

This exploratory study used a qualitative design as part of a mixed-methods study. A partially mixed concurrent equal status design was used, where quantitative and qualitative data were collected in parallel to address the same overarching aims and hypotheses [24]. The rational for this approach is that this design is complementary and seeks to gain a deeper, richer, and more complete understanding of a phenomenon by utilising the different methods to investigate either overlapping phenomena or different aspects of a single phenomenon [25]. This paper draws on the findings from the qualitative component of the study.

The study was approved by the Newcastle and North Tyneside Research Ethics Committee. All participants provided written informed consent and had the capacity to give informed consent. Participants were invited to take part by a letter and detailed information sheet informing them of the aims of the study, that participation was voluntary, they could withdraw at any time, and that data would be treated confidentially. Any identifiable information was removed from transcripts, such as first names or surnames mentioned during interviews, to ensure the anonymity of the interviewee.

### 2.1. Sample

All participants lived in the North East of England and were interviewed between October 2012 and January 2015. Inclusion criteria for people with PD were as follows: a diagnosis of PD made by a movement disorders expert and fulfilled Queen's Square Brain Bank criteria [26] and required an informal caregiver to help with daily activities. Informal carers were spouses, partners, adult family members, or friends who were the primary caregiver of the person with PD.

Purposeful sampling was used to ensure a range of participants and balance between people with PD disease without cognitive impairment, people with PD and mild cognitive impairment, people with PDD, and PD disease severity. Informal carers were sampled based on the PD diagnosis of the care recipient. These three groups were chosen to explore the impact of PD and cognitive impairment across the different diagnoses and to explore whether there were differences in coping and adjustment between groups. PD participants were diagnosed as mild cognitive impairment using Movement Disorder Society Level II classification [27] based on their results on the neuropsychological tests, as described by Yarnall et al. [28]. PDD was diagnosed using Movement Disorder Society criteria [29].

### 2.2. Data Collection and Analysis

For consistency, all interviews were carried out and analysed by the same researcher (RAL), a research assistant and PhD student working on the study and known to the PD participants and carers. One-to-one interviews were carried out at the research unit so that both the PD participants and their carer might speak more freely about their experiences. Interviews were recorded on a digital device. Semistructured interviews were used to allow the researcher to cover the issues or topics relating to the aims of the study in-depth while enabling them to also explore novel subjects or themes as they arose. An interview schedule was developed by RAL, with guidance from KRB, based on a review of the literature (Table 1).

**Figure 1:**
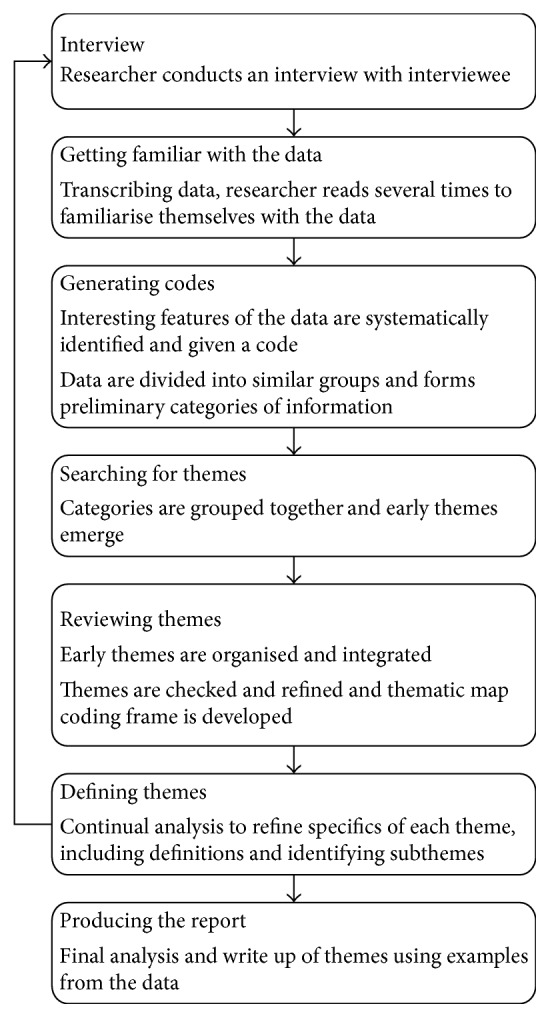
Phases of thematic analysis described by Braun and Clarke [30].

NVivo 11 software was used to aid analysis. The interviews were transcribed verbatim and these texts formed the basis for analysis. Data collection and analysis was an interactive process and continued until saturation was reached, and RAL and KRB agreed no new themes were emerging from the data. Additional interviews were conducted to ensure an equal distribution of participants between the three cognitive groups and to confirm data saturation. The interview content was analysed using thematic analysis as detailed by Braun and Clarke [30] in Figure 1. Open coding by RAL and KRB was at first used in detail, which allowed the early formation of themes and concepts; RAL was the primary coder. Each code was analysed across participants by reviewing all extracts relating to that code; this allowed for a deeper understanding of the codes. A coding frame was derived from the data that included subthemes (Table 2); DC, JPT, and DJB reviewed these conceptually. Differences or inconsistencies in coding and concepts were discussed and were resolved by consensus. The coding frame was used to identify and collate extracts from the interview transcripts from each theme derived from the data [30]. Themes were interpreted in consultation with theory of coping and adaptation [19]. Extracts were reviewed and interrogated as to how they related back to the aims of this study. Selected quotations supporting the analysis and interpretations of results were included in this report.

Rigour of data analysis was ensured by several means. The research team comprised multidisciplinary members, a health psychologist (RAL), a clinical neuropsychologist (DC), an old age psychiatrist (JPT), a neurologist (DJB), and a social gerontologist (KRB). This allowed for investigator and theoretical triangulation of data analysis and interpretation. We referred to consolidated criteria for reporting qualitative studies (COREQ) [31] and reflected on these to ensure methodological rigour and trustworthiness.

## 3. Findings

Thirty-six people were interviewed comprising 18 people with PD and 18 carers (Table 3). The majority of PD participants were male; most caregivers were spousal, with one carer being an adult child of the person with PD and another who was an acquaintance of the person with PD who became an informal carer through mutual agreement.

The qualitative analysis of the participants' interviews revealed a number of themes. Four principal themes emerged in relation to the aims of this study: (1) cognitive impairment as a threat to perceived identity and role, (2) predeath grief and feelings of loss in carers of people with PD and cognitive impairment, (3) success and challenges to coping in people with PD, and (4) problem-focused coping and finding meaning in caring. Additional quotes to support these findings are presented in Supplementary Table 1.

### 3.1. Cognitive Impairment as a Threat to Perceived Identity and Role

This theme related to challenges coping with cognitive impairment in PD. Having PD and cognitive impairment was a threat to participants' perception of themselves and their role within familial and social groups, which was a source of distress.

Participants with mild cognitive impairment and PDD discussed how they were not the same person and that their image of themselves had changed as a result. Being diagnosed with PD to many participants was a life-changing event that split their lives into “before” and “after.” The uncertainty of PD and the awareness of how they had changed physically and mentally, from challenging day-to-day tasks they previously took for granted, were frequent reminders of PD. Their previous self-image was no longer congruent with their physical and mental state, which forced them to construct a new self-image which incorporated PD. Participants grieved for the part of themselves that PD took away.

*I almost had a breakdown because I couldn't remember who I used to be, I literally couldn't, I found that whatever was going on in my life it all revolved around Parkinson's, even when I dream now I've got Parkinson's, to me it's as if it's always been like this … I think what a lot had been wrong with me too was I think I was grieving for somebody I'd lost, which was myself, I found a lot of similarities in grieving the things that you feel.* (Steve, man with Parkinson's and mild cognitive impairment)

For some people with PD, the threat to their perceived identity came from a change in their perceived role in their family. Participants with PD and cognitive impairment felt unimportant or less significant compared to before they had PD. They described feeling redundant and that their roles had been assumed by someone else, often the caregiver. As a result, their self-confidence was diminished, and they felt dissatisfied with their new perceived role within the family environment. The feeling of not being in control of their lives, or control being taken from them, was identified as contributing to this change in identity.

*I used to you know I felt head of the family, not that that means, I didn't wield a stick but you know I couldn't I could never do that, but I felt as though I had a position in the family, but now I don't, I feel downgraded a bit, whether that's paranoia setting in or not I don't know but I just feel a lesser person … I feel as though she's the boss now, really and it's quite rightly is too because she's got me to put up with, so there you are.* (Kevin, man with Parkinson's and mild cognitive impairment)

In contrast to the people with PD and cognitive impairment, those without any cognitive problems did not experience the same threats to their sense of self. They did not describe changes in their perception of themselves, their relationship with their partner, or their place in the family unit. Instead, they described fewer threats to their emotional equilibrium from illness stressors:

*… there wasn't sort of a dividing line where that's pre-PD and post-PD it seemed to be from my point of view it seemed to be a gradual thing … I know I've been diagnosed but nothing dramatic has happened, I think that's what I've been trying to tell the tale throughout I can't see any dramatic change yet.* (David, man with Parkinson's)

Change in role of the carers was also a key issue. Becoming a carer was something they had to incorporate into their own identity and sense of self, although some saw it part of their spousal duty. Part of this was driven by an increase in responsibilities in terms of physical tasks, such as housework, gardening, getting dressed, talking on the telephone, and driving. Carers of people with PD and cognitive impairment also had additional caring activities and responsibilities due to the care recipient not having the cognitive capacity to carry them out, for example, handling household affairs, finances, and decision making. For many, this was a reversal in roles they previously had with PD participants. Carers also perceived their needs were less important than those of PD participants, particularly those with cognitive impairment, and assumed the role of person in charge and protector, which reinforced the role change.

*I never had children and I never wanted children but now I have two children. i.e., my mam and dad … I think that's probably one of the hardest things is that your parents don't become your parents you become the parent and they become the child.* (Liz, daughter of woman with Parkinson's dementia)

### 3.2. Predeath Grief: Feelings of Loss in Carers of People with Parkinson's and Cognitive Impairment

For carers, dissatisfaction centred on changes in their perceived role and feelings of loss. Carers found that cognitive changes in the person with PD were more challenging to deal with emotionally compared to the physical changes. Some carers, predominantly those with partners with PDD, spoke of feelings of loss and grief, both in terms of who the person with PD was and the change in life expectations. Perceptions of people with PD were altered by dementia; a key issue was how the person with PD was no longer themselves, particularly those who also had dementia.

*He was just totally different, he just you'd think he'd been unplugged, it's the only way I could describe it, he was it just wasn't Nigel at all, he was totally different.* (Joyce, wife of man with Parkinson's dementia)

Cognitive impairment had a bigger emotional impact on carers compared to the physical impact of the motor symptoms. The grief centred on how PD and cognitive impairment was taking their loved one from them; some carers referred to that person as already gone. The person they were left caring for was someone they did not recognise. The sense of emotional loss in carers was evident, owing to a mourning of their planned future, restricted social life, and their relationship with the person with PD.

*I suppose they make me feel a little bit depressed at time because I feel as if I'm losing him a bit … I know we've still got a wonderful relationship we've always had a great relationship, sometimes I feel, oh it sounds horrible, as if I'm love, living with an old man which has never been Colin, so I do get depressed over it yes.* (Mary, partner of man with Parkinson's dementia)

For some carers, it was the loss of mutuality as a result of cognitive impairment in PD. The grief encompassed interpersonal loss for their partner, where they had become the sole carer rather than having a mutually beneficial relationship. In addition, the carers had lost their source of social support; they provided increasing support for the person with PD while having no support to cope with their own feelings of anxiety, grief, and powerlessness.

*It's that change from that dual partnership of having that rock beside you to being the carer really and it changes the balance obviously in the relationship which you will know from everybody else, that's hard actually to deal with and try, not to mask it but to try, you use up a lot of energy trying to make things better, to try and regain that balance, you know, and sort of refocus it, to try and help self-esteem and keep trying to keep, almost maintain the status quo, but it's not.* (Barbra, wife of man with Parkinson's dementia)

Only one carer whose husband had PD but no cognitive impairment expressed similar feelings. Although there were no cognitive problems, apathy and depression in the participant with PD were barriers to mutuality and perpetuated feelings of loss and emotional distress in the carer.

*I feel like I've lost my partner, he's become very self-absorbed, it feels like life revolves around him now, it feels like he's living so much in a bubble that he has stopped noticing how things impact on me completely... I actually just feel really lonely … I can't quite find the words to describe how much it's changed our relationship really, I feel like I've lost him in lots of ways (Crying).* (Heather, wife of man with Parkinson's)

### 3.3. Success and Challenges to Coping in People with Parkinson's Disease

This theme centred on the issues around coping and adjustment to PD and cognitive impairment in participants with PD. Some participants coped and adjusted to having cognitive impairment and/or PD better than others. This theme explored the facilitators and barriers to successful coping. Participants with PD and no cognitive impairment generally showed good psychosocial adjustment. They used cognitive and behavioural strategies that facilitated good adjustment when faced with PD-related challenges, such as seeking social support, acceptance of their illness, appropriate expression of emotions, and maintaining their activity levels despite the possible limitations due to having PD.

*I can talk about it now, I found that I couldn't talk about it when I got diagnosed I don't know why like, it's just I didn't want to talk about it as well like you know, but now I can talk to people about it like you know and mainly to say look I've got Parkinson's but I get on with life and I'm doing fine.* (Harry, man with Parkinson's)

Some participants with PD and cognitive impairment had coped better than others. Those that showed good psychosocial adjustment used problem-focused coping strategies to help overcome symptoms of cognitive impairment. Alarms or pill boxes were used to help participants regulate their own medication; some used calendars regularly to remind them about important events and appointments. These coping methods allowed them to maintain some of their activities and independence; it was evident that participants who used problem-focused coping strategies had accepted changes, both physically and cognitively, and had some perceived control over these changes.

*I've got to write on the calendar or, or I just wouldn't I just wouldn't attend like half of them I wouldn't be here … and I'm always checking them to see if I've put the right dates on like you know, just checking and checking and checking just to make sure.* (Rob, man with Parkinson's dementia)

In contrast, some participants with PD and cognitive impairment found coping challenging. For some, their future had changed from something they had control over, to something that was unknown and uncertain. This was difficult to adjust to for some participants. The negative illness representations that some PD participants had, particularly concerning dementia and the possibility of developing dementia in the future, were barriers to high positive affect. Instead, these participants were distressed and had low positive affect:

*…the dementia frightens me more than Parkinson's I think, Parkinson's I've got sewn up, well I'm not going to say sewn up, the other one is the unknown quantity really.* (Nigel, man with Parkinson's dementia)

Rather than using an appropriate expression of emotion, some PD participants vented their emotions. Participants talked about how their condition was unfair, that they felt bitter and angry. This was partly driven by perceived helplessness or low perceived social support. Participants were frustrated by the necessity of being cared for. Some participants described how they “bottled things up” and did not feel they were able to talk to their family about their feelings and the difficulties they experienced. Sometimes this was because they did not want to worry their loved ones, whereas other individuals described themselves as having always been less inclined to seek social support. These participants had low perceived social support and repressed their emotions.

*Sometimes I say, “you take all my independence away from me,” the little bit I try to build up because he is a mother hen, he likes to do things and he thinks he is doing right, I say, “you are not Jack, you are not helping me by being like this,” I feel like I am fighting him and I am fighting the Parkinson's and it is just a vicious circle.* (Denise, woman with Parkinson's and mild cognitive impairment)

PD and cognitive impairment was a disruption to everyday activities in several participants. Some participants had reduced or even avoided activities where they perceived difficulties or potentially distressing situations. The apprehension of being seen as stupid or less than they once were by others was upsetting for some participants, which perpetuated their wish for avoidance or people or certain situations.

*I'm told I am nobody notices but it's knocked my confidence for going up to the shops, going into the town a little bit on my own, and then I worry about getting the money getting out of the, I go shopping and I can pick say a dress up or some shoes and then I start to worry about getting to the counter and getting my money out and getting my card out, I feel as if I'm being slow.* (Sylvia, woman with Parkinson's dementia)

A small number of participants had dysfunctional cognitions and bias. These participants would catastrophize, where small issues with memory or their future would escalate into serious issues. This can be a barrier to good psychosocial adjustment. These participants had low self-esteem, low positive affect, and negative illness perceptions.

*Well, I get frustrated with myself, the worst part about it, as you know, is I'm losing stuff, I just lose so much now, I mean money glasses keys, you name it, they'll just disappear in thin air, that upsets me a bit because I worry about doing something that's going to harm others, I might forget to turn the gas off and leave the water running and stuff like that, that bothers me, as I say.* (Steve, man with Parkinson's and mild cognitive impairment)

### 3.4. Problem-Focused Coping and Finding Meaning in Caring

Coping and adjustment varied between the three groups of carers. Most carers who cared for someone without cognitive impairment had good psychosocial adjustment, where they were able to cope well with illness stressors. Carers of participants with PD without cognitive impairment were able to be more independent that those who cared for someone with PD and cognitive impairment. These carers had increasing responsibilities due to physical disability in the person with PD; however, they identified the importance of not sacrificing their own needs to those of their partner or family member. This was partly motivated by the awareness that the well-being of their partner or family member was dependent on the maintenance of their well-being. Respite from caring facilitated coping in these carers as they were able to rest, seek social support from other sources, and time to gain fresh perspective through the implementation of problem-focused coping strategies.

*I end up doing a lot of things by myself as well because I want to see things too and I'm not going to sit back … I would say no this is my life as well, I've still got to keep it you know otherwise if I go and anything goes wrong with me then Kevin would be in a worse state than ever, so I've got to look after my own interests too without being seen to be selfish you know.* (Anne, wife of man with Parkinson's and mild cognitive impairment)

In contrast, there was tension between the needs of carers and coping with cognitive impairment and PD in their partner or family member. Carers of participants with PDD found it more difficult to cope well with cognitive impairment, and to a lesser extent some mild cognitive impairment carers also found good psychosocial adjustment difficult. These carers had high perceived stress, perceived helplessness, and repressed their emotions; these manifested as self-blame, wishful thinking, venting of negative emotions, and catastrophizing. As a result, carers felt unsupported, isolated, and unable to cope:

*the Parkinson's you think, a piece of cake, the dementia is the real tricky one actually it's that rollercoaster with the dementia … I blame him and that's really not coping because it's not his fault, and I know it's not his fault and I say to myself, “it's the illness,” but sometimes still you want to blame him for your change in life and that's completely wrong and cruel. (Crying) So that's really not coping and, you know, just being less than kind, you feel horrible.* (Barbra, wife of man with Parkinson's dementia)

In addition to increased responsibilities as a result of physical and cognitive disability, there were challenging symptoms and behavioural problems that they also had to cope with. Hallucinations, delusions, and outburst of aggression were not recognised by the person with PD; however, they were evidently distressing for the carers and challenging to cope with. These were very salient intrusions to the lives of the carers and caused disruptions which were unpredictable and distressing. This was exacerbated by carers' feeling they were helpless to both cope with these symptoms and that the person with PDD was not aware of the distress they were causing their partner. This made it difficult for carers to implement problem-focused coping strategies:

*…there's nothing much I can do about it, you see it's difficult because say if you hurt your foot I could help you with your foot and say oh I'll put a bandage on or whatever but if it's something mental the mind you don't know how to cope with that, you can talk to and I'm talking to you I can talk to her and sometimes you see well it's not getting through to her you know because it keeps happening all the time … how do you cope with that, you know?* (Owen, husband of woman with Parkinson's dementia)

Conversely, some carers found meaning and purpose in being a carer, which facilitated good psychosocial adjustment and coping. There was increased appreciation for their family member and expressed an enrichment to their lives from caring. Carers who found meaning and purpose exhibited positive personal growth, where they became more patient, understating, and resilient as a result of caring for their loved one. They further observed gains in the relationship, where the relationship improved with the person with PD.

*When she's come out the shower and I wrap her in the towel and I dry and she's like, “oh you're angel I don't know what I'd do without you,” and that's like more than if you'd won the lottery to be honest … it makes you a better person, that's what I believe, it's made me a better person, it's enriched my life, yeah without a doubt.* (Liz, daughter of woman with Parkinson's dementia)

## 4. Discussion

This qualitative study is the first to explore the impact of cognitive impairment in people with PD and their carers. The findings highlight that for some participants with PD, cognitive impairment negatively impacted on their quality of life and caused emotional distress, but this was not the case for all. It seemed that if PD participants did not have an awareness of any cognitive impairment, and it was not intrusive to their daily lives, then their emotional equilibrium was not disturbed [19]. In carers, however, we found that cognitive impairment has a greater emotional impact than the physical symptoms associated with PD. Central to emotional distress in carers were feelings of loss of their loved one, helplessness, and feeling overwhelmed by cognitive impairment and associated symptoms.

While recent quantitative studies have found that cognitive impairment is associated with poorer quality of life in both patients and their carers [2, 3, 32, 33], they have been unable to determine the impact it has on individuals and how they cope with its effects. We were able to explore this in our study. Our data showed that cognitive impairment threatened emotional equilibrium [19] and affected a range of aspects of their daily lives: social participation, leisure activities, independence, daily activities, mood, and identity.

Challenges to identity and perceived role were important issues, where participants described feeling less confident or insignificant, and PD patients reversed roles with their carer. Through living with PD and cognitive impairment, their previous self-image and social identity were no longer congruent with their current physical and mental state; PD participants suggested that periodic deteriorations caused a crisis which disrupted their emotional equilibrium [19, 34]. Such crises have been proposed to trigger a grief-like mourning period, where individuals grieve for the person they were and their past life before the disease [35]. Parallels can be drawn from other chronic diseases, where this has been coined as ‘chronic sorrow' [36] and has been observed in a previous study in PD [15]. Comparably, role reversal and dissatisfaction has been described previously in carers and people with dementia [37] but has not previously been investigated in PD. Previous studies have illustrated that people with PD who have to accept care and support can feel humiliated and excluded, as well as excluded socially and misunderstood, while partners who were the source of support felt neglected and isolated [12]. This suggests that needing support and becoming that source of support is a potential stressor and could result in poor psychosocial outcomes without the implementation of successful coping strategies [19].

We have extended existing knowledge of cognition and coping in PD. Our findings show that periodically, all PD participants described coping difficulties including uncertain futures, depression, worry, fear, guilt, and anger, which are indicative of poor psychosocial adjustment [19]. Those with cognitive impairment found successful coping difficult and used unhelpful strategies such as wishful thinking, catastrophizing, venting, and negative illness representations; these have been described as emotion-focused strategies by the stress and coping model of adjustment [38]. PD participants without cognitive impairment used problem-focused coping to implement cognitive and behavioural factors to successful adjust more often than those with cognitive impairment [38]. This may be due to participants with PD having insufficient cognitive reserve to implement appropriate coping techniques to facilitate good psychosocial adjustment. Cognitive reserve has been previously suggested as necessary to instigate effective coping strategies [21]. Two quantitative studies proposed that mild cognitive dysfunction could impair the implementation of helpful coping strategies [22], causing difficulties with positive reappraisal, goal setting, and adjusting expectations [23].

We found that carers of people with PD and cognitive impairment experienced greater emotional distress due to the cognitive symptoms compared to the physical symptoms; carers of PDD participants expressed the greatest emotional distress and coping difficulty. Parkinson's dementia carers also described feelings of predeath grief towards their spouses or partners with cognitive impairment, where the person with PDD is no longer cognitively or emotionally present, which was emotionally upsetting for carers. They also grieved for their change in circumstances and for the loss of their planned futures. Predeath grief, also referred to as latent grief or social death, has previously reported that cognitive change was the biggest predictor of carer grief in PD [39].

Parkinson's dementia carers in our study described feeling overwhelmed with cognitive changes and challenging behaviours associated with delusions and hallucinations. Neuropsychiatric symptoms in PDD have previously been associated with increased carer distress [40], similar to the findings of our study. A qualitative study by Williamson, Simpson, and Murray [13] conveyed diverse ways in which spousal carers and people with PD adapted to neuropsychiatric symptoms, with varying success and distress. We found carers expressed difficulty coping and adjusting to hallucinations and delusions our study in comparison to Williamson et al. [13]. Perhaps, this is because the participants in our study were more recently diagnosed with PD, and neuropsychiatric symptoms had first occurred relatively [19].

Our study found that a good mutual relationship between the carer and the patient was protective, with some carers suggesting that they experienced positive personal growth as a result of caregiving. Comparisons can be found in dementia studies; Netto et al. found informal familial carers of people with dementia exhibited positive personal growth, where they become more patient, understating, and resilient as a result of caring for a loved one [41]. Our participants further observed gains and improvement in the relationship with the care recipient. Lyons et al. showed that mutuality was protective of role strain in carers over a 10-year period [42]; it has also been associated with better mental health, lower carer burden, and better carer quality of life [43].

The strengths of this study include the comparatively large number of participants, rigorous methodology, and purposefully sampled participants using theoretical criteria to give a representative sample of carers and people with PD across cognitive groups. Extensive quotations have been presented to ensure the credibility of the analysis. We have also drawn parallels of our findings from other chronic diseases, which denote the validity of our findings. This study was part of a larger mixed-methods study and was able to address the disparities that the quantitative arm of the study. There are several limitations to this study. First, the gender ratio of PD participants and carers were both uneven, with more male PD participants and more female carers taking part. However, this reflects the reality of the disease, where proportionally more men are diagnosed with PD, and that society relies on female care provision [44, 45]. Similarly, most of the carers were the spouse or partner of the person with PD; thus, the experiences of other relatives, such as adult children as carers, are underrepresented. Finally, only PD participants were used in this study; it would be of interest to include carers and patients from other neurodegenerative diseases, such as Alzheimer's disease. Future studies should include a wider range of relationships to the care recipient and should also compare the experiences of patients and carers with different types of dementias.

## 5. Conclusion

This study has highlighted that coping and adjustment to PD and cognitive impairment varies among patients and their carers. Cognitive impairment can threaten an individual's self-perception and their perceived role among family and friends. However, the ostensible effects of cognitive impairment and PD depended on the impact individual's perceived cognitive impairment to have on their daily lives. For carers, cognitive impairment in their partner or family member had a greater emotional impact compared to the physical symptoms of PD, where carers experienced predeath grief. The discussion which developed around protective factors provides possible opportunities for future psychological interventions, such as therapies to improve mood and successful adjustment. By promoting protective factors to enhance existing coping mechanisms, better quality of life for both carers and the person with PD may be achieved to prevent longer term decline and untimely nursing home placement.

## Figures and Tables

**Table 1:** Semistructured interview schedule.

Life before you/they were diagnosed with Parkinson's.
Experience of diagnosis.
How have thins changed?
What does quality of life mean and has this changed?
Discussion on cognitive problems (memory/making decisions/concentrating).
Understanding of dementia in Parkinson's. Discussion on coping.
What helps you to cope with these changes?
Discussion on relationships.
Feelings about the future.

**Table 2:** Coding frame.

Main themes	Subthemes	Descriptors
Cognitive impairment stressors	Cognitive impairment as a threat to perceived identity and role	Loss of sense of self
		Parent/child relationship
		Diminished
		Becoming a carer
		Loss of partner
	Predeath grief: feelings of loss in carers	Not the same person
		Taken from me
		Loss of mutuality and marriage

Coping and adjustment	Success and challenges to coping in people with Parkinson's disease	Successful adjustment
		Acceptance: I just cope
		Maintaining independence
		Uncertain future
		Perceived helplessness
		Avoidance
		Catastrophizing
	Between helplessness and survival in carers coping with cognitive impairment	Sacrificing own needs
		Isolation
		Struggling with cognitive symptoms
		Finding meaning

**Table 3:** Demographics of participants with Parkinson's and their carers.

	People with Parkinson's disease				Relationship with carer	Carer			
	Age	Sex	Occupation	Pseudonym		Age	Sex	Occupation	Pseudonym
Parkinson's disease	62	Male	Retired engineer	Harry	Partner	66	Female	Retired care assistant	Pamela
	69	Male	Retired housing director	David	Spouse	70	Female	Homemaker	Evelyn
	74	Female	Retired dinner lady	Claire	Husband	74	Male	Retired electrical engineer	Frank
	75	Male	Retired air traffic controller	Will	Spouse	67	Female	Retired air traffic controller	Heather
	80	Male	Retired medical lab technician	Mike	Spouse	69	Female	Retired nurse	Kate
	81	Male	Retired doctor	Peter	Spouse	78	Female	Retired radiographer	Ruth

Mild cognitive impairment	65	Male	Retired HGV driver	Ted	Spouse	64	Female	Retired physiotherapist	Opal
	66	Male	Retired solicitor	George	Spouse	61	Female	Solicitor	Frances
	66	Male	Retired nurse	Steve	Carer/friend	—	Female	Retired PA	Nina
	67	Female	Retired catering technician	Denise	Spouse	71	Male	Retired maintenance electrician	Jack
	67	Male	Semiretired sound recording engineer	Edward	Spouse	64	Female	Audio services	Val
	68	Male	Retired salesman	Kevin	Spouse	67	Female	Retired psychiatric nurse	Anne

Parkinson's dementia	67	Male	Retired fitter	Nigel	Spouse	66	Female	Retired shop assistant	Joyce
	70	Female	Retired lab technician	Sylvia	Spouse	77	Male	Retired boiler plater	Owen
	72	Male	Retired labourer	Rob	Spouse	72	Female	Retired care assistant	Gwen
	79	Female	Retired shop assistant	Ingrid	Mother/Daughter	52	Female	Carer	Liz
	69	Male	Retired local government officer	Alan	Spouse	56	Female	Teacher	Barbra
	87	Male	Retired salesman	Colin	Partner	68	Female	Retired	Mary

## Supplementary Materials

Supplementary Materials Supplementary Table 1: additional quotes from the thematic analysis of people with Parkinson's and their carers.
