# Supplementary material for: Coping with Cognitive Impairment in People with Parkinson's Disease and Their Carers: A Qualitative Study
Source: Parkinsons Dis. 2018 Apr 8;2018:1362053. doi: 10.1155/2018/1362053 (PMC5911314; doi:10.1155/2018/1362053)
Supplement: Supplementary Materials — Supplementary Table 1: additional quotes from the thematic analysis of people with Parkinson's and their carers. [file 1362053.f1.pdf]

Supplementary Table 1: Additional quotes from the thematic analysis of people with Parkinson's and their carers

Theme	Quotes
Cognitive impairment as a threat to perceived identity and role	I was independent, I liked driving, I liked going out with the lasses from work and I don't do any of that now, I liked swimming and I don't do that now… I feel that since I got Parkinson's I have lost something and I can't get it back, it is hard to describe how I feel in that way, I felt angry when I first got it because I thought, why have I got it? I can't think of anything more to say, my life has changed a lot since I got it (Denise, woman with Parkinson's and mild cognitive impairment)<br><br>…they don't let me do very much, they think I should just sit in a chair and watch them and not worry about whether the shopping gets done or the housework gets done or the shopping gets done, cause they're there and they're in charge and I'm not (Ingrid, woman with Parkinson's dementia)<br><br>I think we've reversed roles, I've gone from being spoilt, looked after, always have been, and Nigel did most things and whatnot, and I saw it as if I have to do everything now, and I have to, I don't tell him everything where before we always told each other everything, you know like something with the family I don't tell him because I think eeh he'll just worry about that, he'll just get you know it'll, so I keep a lot to myself (Joyce, wife of man with Parkinson's dementia)
Pre-death grief: feelings of loss in carers of people with Parkinson's and cognitive impairment	I think it's taken the man I married, the man that I thought I was going to spend my old age with, the life we planned which we had it all planned, it's gone, and Parkinson's took it, definitely, at first I was frightened of it I was frightened what it was going to do to us both emotionally and financially, because that made a vast difference to our life when Ted finished work (Opal, wife of man with Parkinson's and mild cognitive impairment)
Success and challenges to coping in people with Parkinson's disease	Well Kate's a great coper, she thinks positive all the time, and she just gets on with it, she won't allow me to be defeatist, you know she makes light of things (Mike, man with Parkinson's)<br><br>It's changed in that looking to the future is not good, in fact a bit bleak at times if I do get upset about the possible prospects… I get a bit miserable, just I mean it's difficult to describe and put your finger on, just I suppose depressed is the only word that easily springs to mind (George, man with Parkinson's and mild cognitive impairment)<br><br>I feel as if I'm a burden I don't want to be a burden… I'm not doing what I should be doing, like I should be doing house work and I should be doing washing and ironing and I'm not (Ingrid, woman with Parkinson's dementia)<br><br>I don't like going to parties where there's people who I ought to know and remember their names because the chances are I don't, Val is more of a party goer than I am, she'll drag me out occasionally, not nearly as often as we used to (Edward, man with Parkinson's and mild cognitive impairment)
Problem focused coping and finding meaning in caring	George got it into his head that the house was falling down and that was a major anxiety for quite a while despite every reassurance from me… but it was a genuine belief at the time, he really was worried that when we got the surveyor round you know to sell the house he would probably find something like the house was subsiding or there was a major crack in it and the whole thing was going to go down like a pack of cards, and at first I just laughed at it but I stopped doing that because it's just a horrible fear (crying) (Frances, wife of man with Parkinson's and mild cognitive impairment)<br><br>I actually think we have a good quality of life, because we enjoy the time we spend together… I think our quality of life has changed, but it's not a bad quality of life that's been left in its place, it's different to what we had planned, but we still do okay (Opal, wife of man with Parkinson's and mild cognitive impairment)
